# A Mesoscale Model-Based Climatography of Nocturnal Boundary-Layer Characteristics over the Complex Terrain of North-Western Utah

**DOI:** 10.1007/s10546-015-0044-6

**Published:** 2015-05-30

**Authors:** Stefano Serafin, Stephan F. J. De Wekker, Jason C. Knievel

**Affiliations:** Department of Meteorology and Geophysics, University of Vienna, Vienna, Austria; Department of Environmental Sciences, University of Virginia, Charlottesville, VA USA; National Center for Atmospheric Research, Boulder, CO USA

**Keywords:** Boundary-layer separation, MATERHORN Project, Mountain meteorology, Stable boundary layer

## Abstract

Nocturnal boundary-layer phenomena in regions of complex topography are extremely diverse and respond to a multiplicity of forcing factors, acting primarily at the mesoscale and microscale. The interaction between different physical processes, e.g., drainage promoted by near-surface cooling and ambient flow over topography in a statically stable environment, may give rise to special flow patterns, uncommon over flat terrain. Here we present a climatography of boundary-layer flows, based on a 2-year archive of simulations from a high-resolution operational mesoscale weather modelling system, 4DWX. The geographical context is Dugway Proving Ground, in north-western Utah, USA, target area of the field campaigns of the MATERHORN (Mountain Terrain Atmospheric Modeling and Observations Program) project. The comparison between model fields and available observations in 2012–2014 shows that the 4DWX model system provides a realistic representation of wind speed and direction in the area, at least in an average sense. Regions displaying strong spatial gradients in the field variables, thought to be responsible for enhanced nocturnal mixing, are typically located in transition areas from mountain sidewalls to adjacent plains. A key dynamical process in this respect is the separation of dynamically accelerated downslope flows from the surface.

## Introduction

**Fig. 1:**
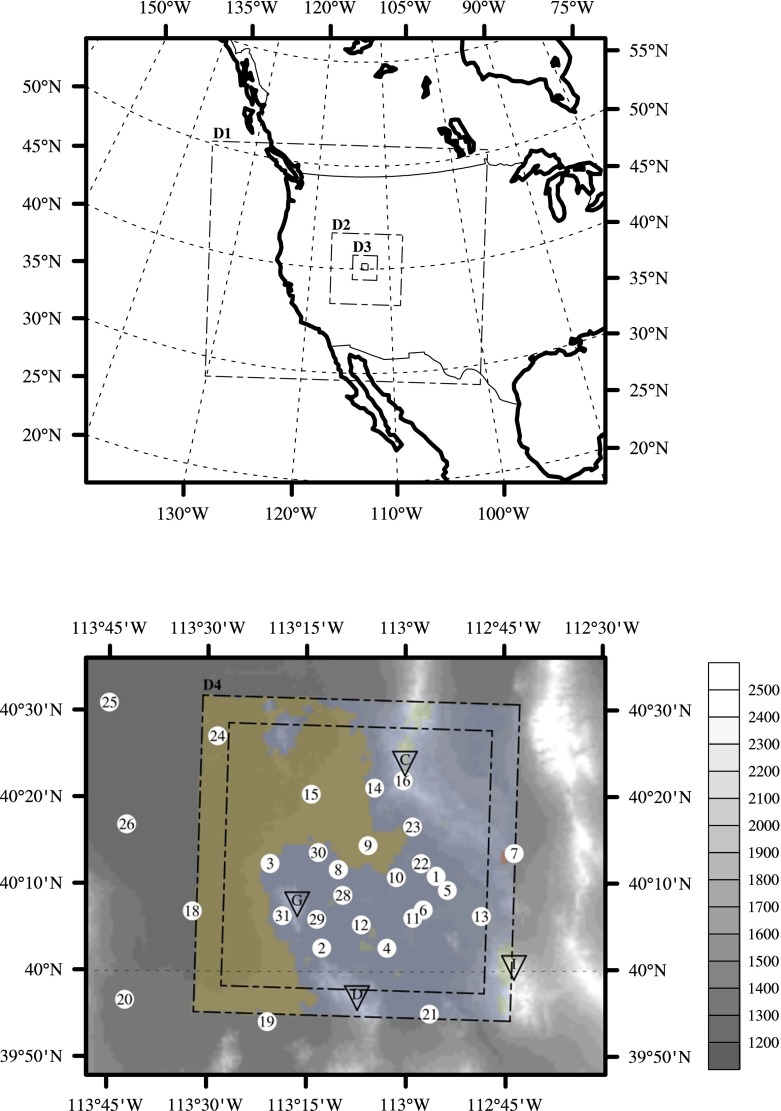
Map of the study area. The *upper diagram* shows the four domains of the 4DWX model simulations in the Dugway Proving Ground region. Below is a zoom-in on the Dugway Proving Ground area, with *grey shading* representing surface altitude in m AMSL and *colours* referring to land-cover categories (*yellow* for playa, *blue* for shrubland). Two squares (*dot-dashed lines*) are drawn in the map. The larger one outlines the boundaries of domain 4 in 4DWX model simulations; the smaller one outlines the part of the domain that is not subject to flow relaxation towards the lateral boundaries. *Numbers in white circles* denote the position of SAMS automatic weather stations (stations 17 and 27, missing in the map, are located farther to the north). *Letters in the down-pointing triangles* refer to the major orography features in the area: Granite Peak (G, 2148 m above m.s.l.), the Cedar Mountains (C, 2110 m above m.s.l.), the Dugway Range (D, 2082 m above m.s.l.) and Indian Peaks (I, 2566 m above m.s.l.)

Granite Peak, located in the Dugway Proving Ground in north-western Utah, is an isolated mountain rising about 800 m above the surrounding terrain (Fig. 1). It has an approximately ellipsoidal shape oriented from north-north-west to south-south-east and its main axes are respectively 10 and 6 km long. A flat salt plain (*playa*) lies west and north-west of the peak, and to the east lies a broad valley (approximately 40 km wide) gently sloping towards the north-west, covered by herbaceous vegetation. This arid grassland is surrounded almost completely by other prominent peaks—the Cedar Mountains to the north-east, Indian Peaks to the south-east, and the Dugway Range to the south-west.

During the night, these topographic features are favourable for the onset of diverse local wind systems in the planetary boundary layer (PBL). In particular the cooling phase of the diurnal cycle generates drainage flows, especially during weak synoptic conditions (Whiteman 2000). Cold air can pool at night in topographically sheltered areas when the diabatic cooling rate imposed by the radiative loss of energy at the ground is strong (Smith et al. 2010; Price et al. 2011). Pools of cold air may also become persistent, i.e., last over multiple days, when synoptic and mesoscale air motions combine with turbulent, radiative, and cloud-scale processes in preventing cold-air pool removal during the day (Lareau et al. 2013).

Besides thermally-driven phenomena, a variety of dynamically forced flows may occur if the atmosphere is stably stratified down to the ground. When the upstream flow conditions support propagating mountain waves, the related local pressure minima on the leeside slopes of mountains contribute to the downslope acceleration of the ambient flow (e.g., Nappo 2013). Dynamically accelerated downslope flows are not necessarily intense and damaging, but they originate from essentially the same type of forcing that causes downslope windstorms at many locations around the world, e.g., the north-eastern Adriatic Sea (Bora winds, Grisogono and Belušić 2009), the foot of Colorado’s Front Range (Lilly 1978), or Owens Valley in California, east of the Sierra Nevada (Grubišić et al. 2008).

Pressure perturbations embedded in mountain waves can be strong enough to force the PBL to separate from the ground near the foot of mountain slopes (Jiang et al. 2007) and, in extreme cases, develop highly turbulent rotors (Kuettner 1959; Doyle and Durran 2002, 2007; Vosper 2004). Downslope flows occurring in conjunction with mountain waves can lead to cold-air pool removal or displacement (Lee et al. 1989; Lareau and Horel 2014), in a process that involves the formation of a non-stationary microscale front and low-level convergence (Lareau et al. 2013).

Atmospheric wakes (Epifanio 2003) and gap flows (Mayr et al. 2007), especially at the rather narrow constriction separating Granite Peak from the north-western tip of the Dugway Range, are among the other phenomena expected to occur in Dugway Proving Ground under statically stable conditions.

Beyond topography, at Dugway Proving Ground also land-cover variability plays a role in generating thermally-driven flows. The most prominent is a diurnally-varying flow from the playa to the sagebrush plain, induced by differences in sensible heat flux between the two regions. In analogy to sea and lake breezes, circulations arising from differential heating in such conditions have occasionally been referred to as “salt breezes” (Physick and Tapper 1990; Rife et al. 2002).

This wide range of possible weather phenomena makes this region an ideal location to further our understanding of multiscale mountain flows. Two field campaigns related to the MATERHORN project took place in the Dugway Proving Ground in autumn 2012 and in spring 2013 (Fernando et al. 2015). Special instrumentation deployed during the project complemented an existing permanent mesoscale network of surface measurement stations (Surface Atmospheric Measurement Systems, SAMS), with an average density of approximately 1 station per 100 km$^2$.

At the Dugway Proving Ground, and in particular near Granite Peak, the interaction between (dynamically forced) downslope winds and (thermally forced) drainage flows may frequently lead to convergence lines between airmasses with markedly different properties. Sonic anemometer and scanning lidar measurements provided observational evidence of these convergence lines during MATERHORN (e.g. Lehner et al. 2015; Fernando et al. 2015). Such events generate vigorous mixing even during the night, in contrast to the typical behaviour of the stable boundary layer over flat terrain (Fernando et al. 2015). These MATERHORN observations have been interpreted almost exclusively as evidence of “collisions” of drainage flows, caused by differential thermal forcing along slopes and adjacent valleys.

We test this hypothesis by analyzing operational high-resolution numerical weather prediction products, which are continuously available for Dugway Proving Ground from the 4DWX model system. The model system was developed by the NCAR Research Applications Laboratory (Davis et al. 1999; Liu et al. 2008) and provides eight daily runs with hourly model output. Simulations from the 4DWX model system are used to build a short-term climatography (2012–2014) of nocturnal PBL phenomena, with a focus on the impact of mesoscale atmospheric processes on the PBL.

We expect our results to be relevant in the context of the MATERHORN project and of the routine operations in Dugway Proving Ground. First, insights from the following analysis can, if necessary, be exploited in designing and implementing field studies. Second, quantitative knowledge of the typical flow phenomena in the area, and of their modulation by topography and land-cover features, is advantageous in the correct interpretation of MATERHORN measurements and in the generalization of results from case studies. Third, information about the typical airflow regimes in the region provides scenarios for idealized simulations (e.g., aimed at understanding specific processes), or for applied studies (e.g., to model pollutant or tracer dispersion).

The paper is organized as follows: Sects. 2 and 3 introduce and compare measurements from the SAMS network and 4DWX model simulations, discussing the prevailing wind regimes at Dugway Proving Ground and the diurnal variability of wind direction at a few selected sites. Section 4 focuses on seeking preferential areas for flow convergence or boundary-layer separation, while discussions and conclusions are presented in Sect. 5.

## Data and Methods

### Observational Data

Near-ground observations are available from the SAMS stations maintained by the Dugway Proving Ground personnel. A SAMS station typically comprises probes that measure temperature and relative humidity at 2 m a.g.l. and vane anemometers that measure wind speed and direction at 2 m and 10 m a.g.l.. SAMS data used in the present study are from the 2-year period between 1 July 2012 and 30 June 2014 and are available as 5-min averages, with data availability exceeding 90 % at most of the 31 stations, except for two that were installed only recently.

### The 4DWX Model Forecast System

Since the 1990s, the Dugway Proving Ground has used a continuously operating meso-gamma-scale analysis and forecast system (4DWX) developed by the NCAR Research Applications Laboratory (Davis et al. 1999; Liu et al. 2008). Largely sponsored by the U.S. Army Test and Evaluation Command, the 4DWX model system is now in use at eight different test ranges in the United States. While the primary use of the modelling system is weather forecasting, coupled applications for pollutant dispersion modelling and noise assessment (Sharman et al. 2008) are also operated. The Dugway Proving Ground implementation of the system is currently based on version 3 of the Advanced Research core of the Weather Research and Forecasting (WRF) numerical model (Skamarock and Klemp 2008). It operates with a grid spacing of 1.1 km in the innermost of four domains and provides weather analyses and forecasts at hourly intervals. Eight forecast cycles are run every day. Simulations starting at hour *t* (real time) are initialized at time $t-3$ and are nudged toward observations during roughly the first 3 h of the run, nominally from $t-3$ to *t*. Observations from even earlier than the start of the nudging cycle indirectly influence a simulation through the previous cycle’s restart files, which are the basis for “hot-start” initializations. The initial hours of all runs, taken consecutively, form a continuous final analysis (Liu et al. 2008) whose data are considered in the present study. The 4DWX model data used herein are from 1 July 2012 to 30 June 2014, and model fields at hourly resolution are available for almost all days during the two years (95 % of the time), the few gaps being due to maintenance or unexpected downtime of the computing infrastructure.

The physics parametrizations for the WRF model in the 4DWX system include the Yonsei University (YSU) PBL scheme (Hong et al. 2006), the Noah land-surface model (Chen and Dudhia 2001), the Monin–Obhukov surface-layer scheme (Janjić 2002), the Dudhia scheme (Dudhia 1989) for shortwave radiation, the Rapid Radiative Transfer Model (RRTM, Mlawer et al. 1997) for longwave radiation, the updated Kain–Fritsch cumulus scheme (Kain 2004), and the Thompson microphysics scheme (Thompson et al. 2004). Explicit sixth-order hyperdiffusion is applied to suppress noise in kinematic fields, especially in the convective boundary layer under weak winds (Knievel et al. 2007).

Two aspects of this model configuration deserve further attention. First, the adoption of a first-order turbulence parametrization scheme (such as the YSU model) may be questioned for applications over complex terrain. First-order closures are generally over-diffusive (Grisogono 2010; Zhang et al. 2013) and are one-dimensional by design, preventing any consideration of turbulence advection effects that may be relevant over mountainous topography (Arnold et al. 2012). Nevertheless, several model intercomparison studies focused on complex terrain applications (e.g., Schmidli et al. 2010; Doyle et al. 2011; Zhang et al. 2013) did not show any significant or systematic difference between the simulations from first-order closure models and those from more sophisticated turbulence parametrizations.

Second, numerical hyperdiffusion, while beneficial for the modelling of the convective boundary layer, may be detrimental for the modelling of nocturnal conditions, in which turbulent diffusion is very low. This aspect of the 4DWX model system reflects its primary use in operational forecasting, for which the onset of non-linear numerical instability (which can be prevented also by means of numerical hyperdiffusion) is a major concern. Some simulations by the 4DWX model system at night might be overly diffusive at small scales, which should be kept in mind when interpreting our analysis.

The 4DWX simulation domains are displayed in Fig. 1: the 1.1-km domain is centred on the sagebrush plain east of Granite Peak, and its horizontal mesh consists of 60 $\times$ 60 grid points. Flow relaxation is imposed within five grid points from all lateral boundaries in order to enforce boundary conditions by means of Newtonian nudging. Consequently, in the area outside the core region the wind field is not in exact balance with the pressure field and the other source terms. The outer “halo region” is therefore discarded in all the analyses presented herein.

## Observed and Modelled Wind Climate

### Comparison Between Observations and Simulations

An exhaustive verification study of 4DWX model simulations is beyond the scope of the present work. Rather, what is of interest here is the model’s ability to reproduce, in a climatological sense, the observed wind variability in the area. Therefore, instead of concentrating on individual days or case studies, we try to determine the extent to which the 4DWX forecast system can reproduce the general character of the near-surface atmospheric circulation, e.g., variability in wind speed and direction. Measurements from a few selected stations in the SAMS network, 5-min averages available every 5 min, are used as the observational counterpart.

**Fig. 2:**
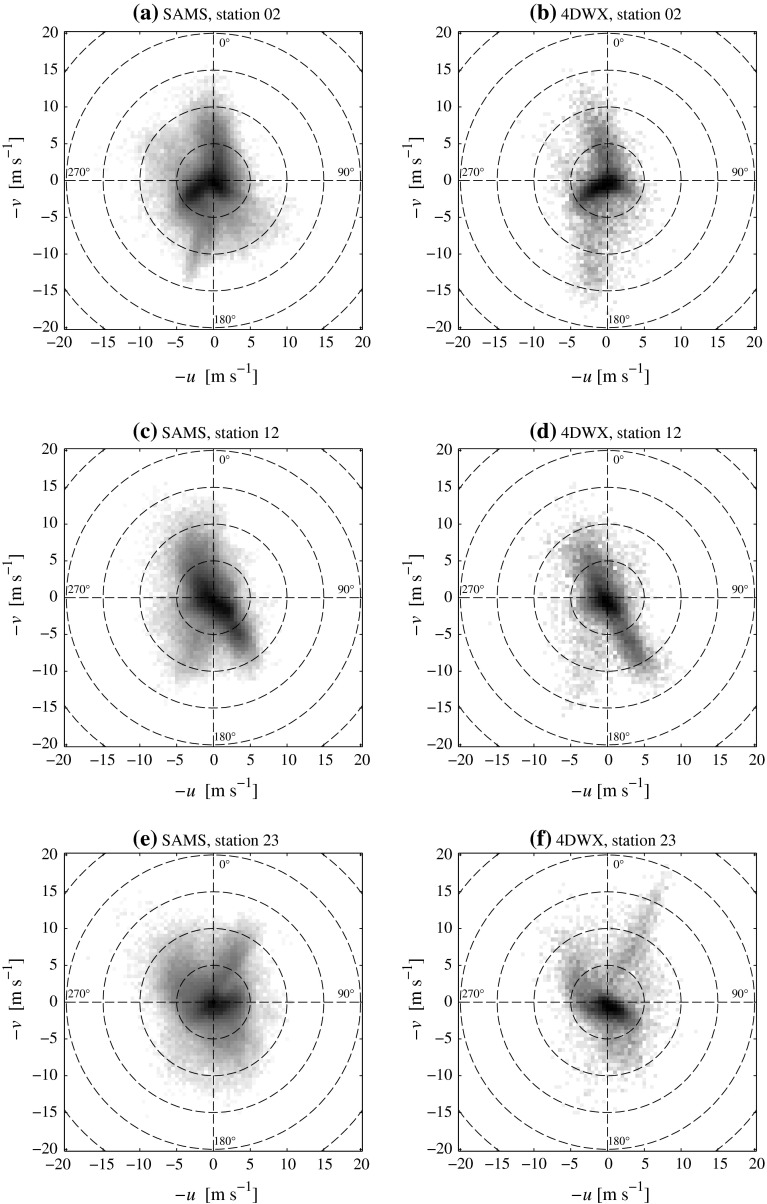
Two-dimensional histograms of 10-m wind components *u* versus *v* at SAMS stations 2, 12, and 23, and at the corresponding nearest-neighbour grid points in the 4DWX model domain. Relative frequencies in each histogram sum up to 100 %. The *grey scale* is proportional to the population of bins and is logarithmic in order to make even rare events clearly visible. Highly populated bins are displayed in *darker shading*

Figure 2 presents joint frequency distributions (i.e., two-dimensional histograms) of the west-east and south-north wind components at three SAMS stations and at their respective nearest-neighbour grid points in the 4DWX model domain. These diagrams provide essentially the same information as wind roses, but allow a better appreciation of flow regimes that are only poorly represented in the sample. For each of the bins in these histograms, the distance from the origin of the diagram identifies the wind speed and direction classes, while the grey shading is proportional to the relative frequency.

The three selected measurement stations are numbers 2, 12, and 23, located respectively in the wide gap separating Granite Peak and the Dugway Range, in the sagebrush plain east of Granite Peak, and at the foot of the south-western slope of the Cedar Mountains. These three locations are prototypical of the different flow patterns expected at Dugway Proving Ground: gap flow at station 2, nocturnal drainage at station 12, and dynamically accelerated downslope winds at station 23. Although not shown here, statistics from other stations were also examined to ensure that the three selected stations are adequately representative. For instance, the nearby stations 4 and 12 have very similar wind direction and speed statistics, even if nocturnal drainage at the valley bottom appears to be more persistent at the former. Similarly, stations 23 and 13 both show signatures of downslope flows (more frequent at the latter), although the local orientation of the slope is different.

The most frequent wind direction at station 2 (Fig. 2a, b) is west-south-westerly (positive *u* and *v* components, direction approximately 240$^\circ$), while wind speeds in this directional range, which corresponds to gap flow into the Dugway Plain, are typically below 5 m s$^{-1}$. Flows with an easterly component, i.e., into the salt plain, are also observed, but generally have a lower wind speed and a somewhat larger directional variability. Large wind speeds, exceeding 15 m s$^{-1}$, are much less frequent and related to completely different prevailing wind directions: southerly (200$^\circ$) and northerly (340$^\circ$–020$^\circ$). These directions do not correspond to the main axis of the gap, but are approximately parallel to the sidewalls of nearby mountains, probably evidence of stable air masses frequently flowing around obstacles.

At station 12 (Fig. 2c, d), the main lobes of the frequency distribution of wind components are elongated from north-west to south-east, along the direction of the gentle valley slope. The most frequent directions are between 090$^\circ$ and 180$^\circ$ (negative *u*, positive *v*), with wind speeds mostly below 5 m s$^{-1}$, likely related to nocturnal drainage or to synoptic-scale southerlies dominant in the area (see below). Relatively stronger winds also seem to be preferentially south-easterly (150$^\circ$), although they can occur from almost all sectors.

Station 23 (Fig. 2e, f), located at the foot of the Cedar Mountains, displays a larger wind-direction variability than the other two stations. Even in this case, the most frequent wind direction (easterly) seems to be related to local modification of the prevailing southerly large-scale flow. Strong winds may occur equally frequently from almost all directions, but with a distinct preference for the north-easterly (030$^\circ$), a possible hint at windstorms down the south-westerly slope of the Cedar Mountains.

Visual comparison between measurements and 4DWX model simulations indicate good agreement in the representation of both the most frequent wind directions and the typical wind speeds. The overall impression is that the set of simulations from the 4DWX model system is a reliable representation of the observed wind climate. Similar considerations apply to almost all other SAMS stations in the area, with a few expected exceptions (as, for instance, station 16 atop Cedar Mountains).

**Fig. 3:**
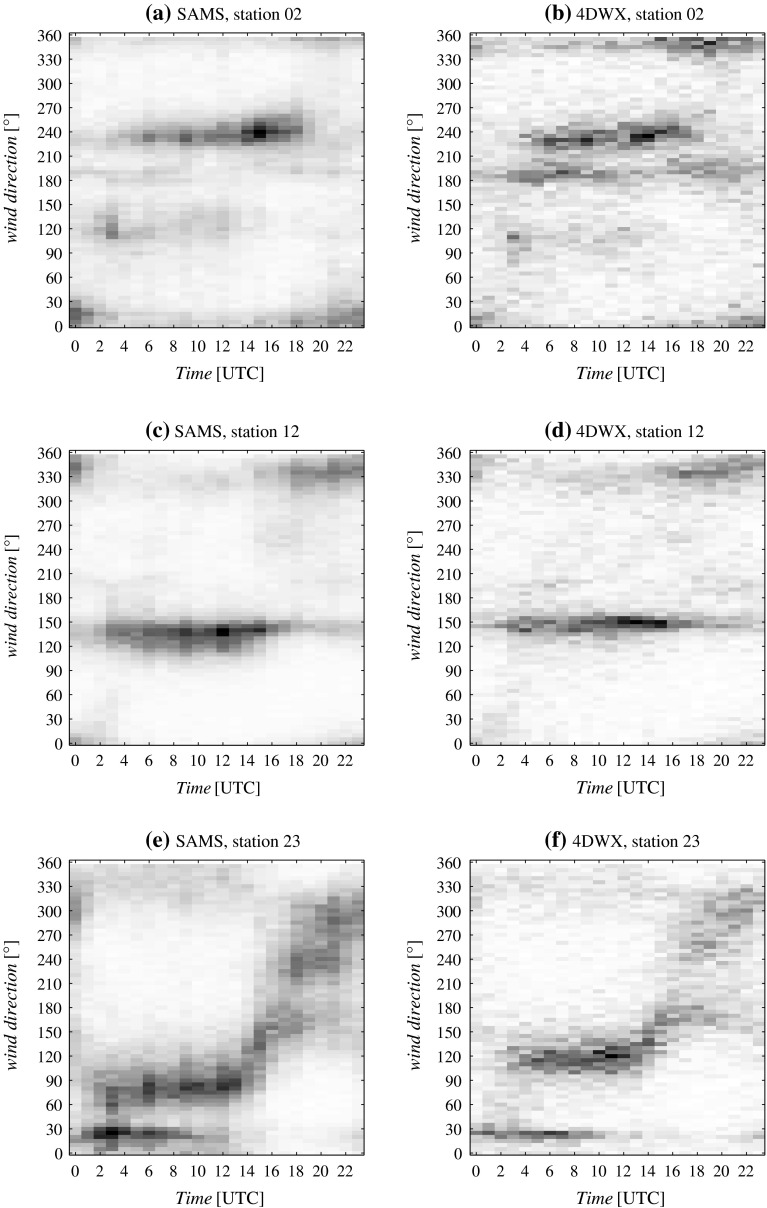
As in Fig 2, but for the frequency of occurrence of 10-m wind speed $>1$ m s$^{-1}$ as a function of wind direction and time of day

Histograms in Fig. 2 do not discriminate between diurnal and nocturnal flow patterns. The diurnal cycle of wind directions at stations 2, 12, and 23, and at the respective closest model grid points, is therefore depicted in Fig. 3. (Only wind speeds $>$1 m s$^{-1}$ are considered, given the poor representativity of wind directions at extremely low wind speeds.) Apart from the clockwise bias in simulations at station 23, the diurnal cycle of wind direction from the 4DWX model system is quite realistic.

Taking seasonal variability into account, nighttime at the Dugway Proving Ground corresponds approximately to the period between 0300 and 1500 UTC, with sunrise and sunset times ranging respectively from 1259 to 1449 and from 0005 to 0303 UTC (from 0559 to 0749 and from 1705 to 2003 local time, LT). At all three stations, the distribution of nocturnal wind directions shows a well-defined primary direction (240$^\circ$ at station 2, 140$^\circ$ at station 12, and 090$^\circ$ at station 23) and a few equally well-defined secondary directions (e.g., 000$^\circ$, 120$^\circ$, and 190$^\circ$ at station 2; 330$^\circ$ at station 12; and 030$^\circ$ at station 23). The primary direction corresponds to the main axis of the gap between Granite Peak and the Dugway Range at station 2, to drainage along the north-west oriented Dugway Plain at station 12, and to eastward deflection of nocturnal southerlies at station 23. Secondary directions at station 2 likely correspond to blocked flow running parallel to either Granite Peak or the Dugway Range and ultimately into the gap, while the secondary direction at station 23 is related to downslope flow from the Cedar Mountains (see Fig. 1).

**Fig. 4:**
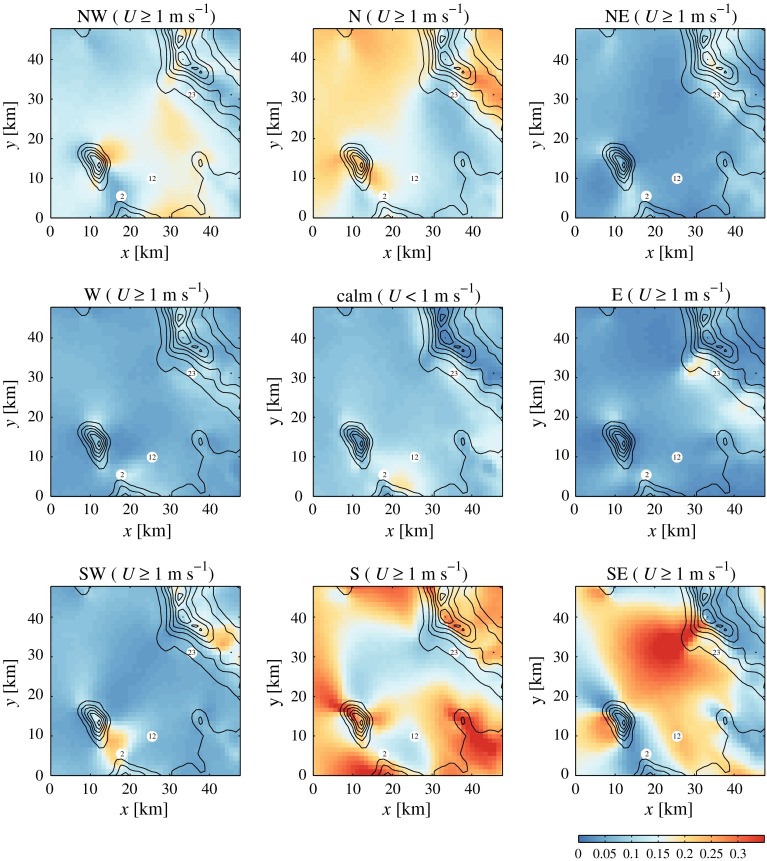
Relative frequency of near-surface wind direction in eight directional sectors in 4DWX model domain 4. Sectors are 45$^\circ$ wide and are centred on directions ranging from 000$^\circ$ (north) to 315$^\circ$ (north-west). Only wind speeds $>$1 m s$^{-1}$ are considered. The frequency of wind speeds $<1$ m s$^{-1}$ is represented in the *middle panel*. At each model grid point, frequencies from the nine charts sum up to 100 %. *Isolines* in all panels refer to surface altitude. The spacing between contours is 100 m. The *colour bar at the bottom left* refers to frequencies in the range [0, 1] and applies to all panels

Because of the large impact that topography exerts on the mesoscale behaviour of cold and stable airflow, the prevalent nocturnal wind directions are sharply defined and seemingly easy to relate to topography features. In contrast, daytime variability in wind direction is larger. Bimodal distributions of wind directions in the afternoon are to a certain degree visible at all stations. The two main lobes of the distributions, which are most clearly visible at station 12, apparently correspond to the north-north-westerly and southerly wind directions typical of the synoptic climatology in the area. Arguably, increased mixing during daytime favours the downward entrainment of momentum from the free atmosphere. A contribution to daytime flow from the north-north-west sector at stations 2 and 12 might also be due to upvalley winds. Gradual clockwise rotation of the wind in the early morning is apparent at station 23, consistent with a morning transition regime and the local onset of upslope winds towards the Cedar Mountains.

Diurnal cycle statistics computed from 4DWX model simulations generally agree well with observations despite minor discrepancies, e.g., the south-easterly rather than easterly direction of nocturnal flows at station 23, or more frequent southerly flows in the night at station 2 (see Fig. 2). The encouraging results of pointwise comparisons between simulations and observations justify looking with confidence at statistics calculated over the whole core area of 4DWX model domain 4.

### Wind Climate Simulated by the 4DWX Model System

The relative frequency with which the 4DWX model system simulates the wind direction from eight sectors is represented in Fig. 4. Sectors are 45$^\circ$ wide and centred on the 000$^\circ$, 045$^\circ$, 090$^\circ$, etc., directions. Only wind speeds $>$1 m s$^{-1}$ are considered and no distinction between night and day is made. The relative frequency of calm conditions is represented in the diagram at the centre of the figure. Southerly (or south-easterly) and northerly (or north-westerly) winds dominate the wind climate in the region. However, topography significantly influences the small-scale variability of the prevailing winds.

While northerly and southerly flows are more frequent over the playa, the dominant wind directions in the wide valley between Granite Peak and the Cedar Mountains are north-westerly and south-easterly, possibly as a consequence of flow channelling and/or thermal forcing. The most frequent wind direction in the gap between Granite Peak and the Dugway Range is south-westerly, i.e., aligned with the main axis of the gap and into the Dugway Plain. Gap flow along the opposite direction also occurs, but much more rarely.

Southerly winds are very frequent on the northern slope of the Dugway Range, but relatively uncommon immediately north of it. This suggests that downslope flows tend to detach from the surface at the foot of the mountain without extending over the plain. Local forecasters often observe a pulse of southerly flow that crosses the plain just after sunset, after which the flow becomes very light or calm and remains so throughout the night (Matt Jeglum, personal communication, 2015). The common occurrence of calm conditions just north of the Dugway Range is confirmed by 4DWX model simulations, as visible in the central panel of Fig. 4. Similar considerations are valid for the northern slope of Granite Peak. Frequent flow separation and related convergence lines are expected at these locations.

Winds from the east and north-east are generally rare, but comparatively more frequent on the south-western slope of the Cedar Mountains. Locally, a spur detaching from the main ridge of the Cedar Mountains steers the predominant south-easterly flows to easterly. Similarly, the frequent south-westerly flows in the gap between Granite Peak and the Dugway Range might be caused by the eastward deflection of locally dominant southerlies.

Flow around Granite Peak is common, as clearly evident in a number of diagrams. For instance, north-westerly and south-easterly flows are more frequent along the north-east and south-west sidewalls of the mountain than along the north-west and south-east ones. Similarly, southerly and northerly flows are more common on the east and west sides of the obstacle than on the south and north ones.

**Fig. 5:**
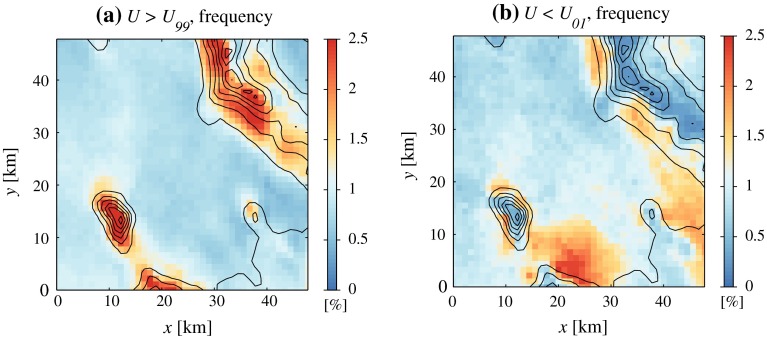
Two-dimensional histograms representing the spatial distribution of extreme wind speeds (highs in *a* and lows in *b*). The frequency of exceedance (resp. not exceedance) of the 99th (respectively 1st) percentile is represented. A spatially uniform distribution of extremes would correspond to a uniform value of 1

As with wind direction, extreme wind speeds appear to be closely connected to topography (Fig. 5). The two panels in Fig. 5 display the spatial distribution of wind speeds respectively greater than the 99th and smaller than the first percentile in the whole Dugway Proving Ground area. High wind speeds are much more frequent at mountain tops than above plains, as expected. However, they are also frequent on mountain slopes (e.g., on the south-west flank of the Cedar Mountains or north-west of Camelback Mountain, the low hill at approximately $x=38$ km and $y=14$ km), suggesting dynamically-induced downslope acceleration.

Extreme wind speeds tend to develop more frequently on the slopes of the Cedar Mountains than of Granite Peak, presumably because of the different vertical aspect ratios of the two ridges. Stable airflow over Granite Peak, which is narrow and steep, falls more easily in the potential flow regime, in which maximum wind speeds occur right at the mountain top (e.g., Lin 2007). Instead, airflow over the Cedar Mountains, which are considerably broader and slightly lower, apparently favours vertically propagating waves and concomitant downslope acceleration.

Low wind speeds (Fig. 5b) are infrequent at mountain tops, while they occur most commonly over the plains closest to mountain slopes, in particular north of the Dugway Range (in agreement with Fig. 4 above). Flow stagnation or convergence at these locations is likely related to downslope flow separation, as explained extensively in Sect. 4 below.

**Fig. 6:**
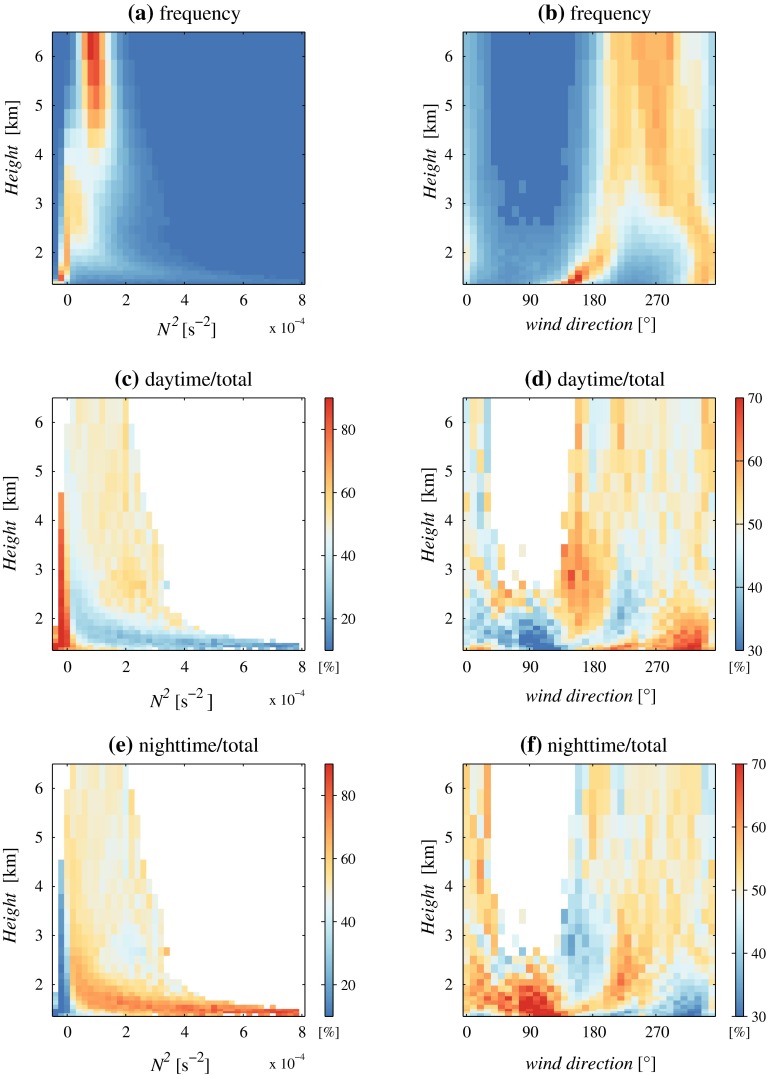
Two-dimensional histograms representing the variability of **a** buoyancy frequency and **b** wind direction with height above m.s.l. Relative frequencies in each of histograms (**a**) and (**b**) sum up to 100 %. Panels **c** and **d** represent the fraction of daytime events with respect to the total in each bin of panels (**a**) and (**b**). Panels **e** and **f** represent the fraction of nighttime events. Corresponding bins in panels **c** and **e** sum up to 100 %. The same applies to panels (**d**) and (**f**). *White areas* in panels **c**–**f** correspond to scarcely populated bins

To gain insight into the typical features of vertical atmospheric profiles in the area, histograms representing the variability of stratification (in terms of the buoyancy frequency) and of wind direction with height, at the centre of 4DWX model domain 4, are shown in Fig. 6. Figure 6a and b shows that stable stratification ($N^2\approx 10^{-4}$ s$^{-2}$) and persistent westerly or south-westerly winds occur in the mid-troposphere, above 4000 m above mean sea level (m.s.l.), as is typical in midlatitudes. Stratification and wind direction vary more at lower altitudes, in particular between 2000 and 4000 m above m.s.l., presumably related to synoptic weather systems. Two distinct branches of southerly and north-westerly winds are in fact apparent, characterized respectively by veering (warm advection) and backing (cold advection) with height. Below 2000 m above m.s.l., within a few hundred metres above ground, both southerlies and north-westerlies have a tendency to veer with height. Since this range of altitudes often corresponds to a well-mixed layer, this latter feature is probably due to the balance established between the pressure gradient force and frictional and rotational effects (Ekman spiral). While well-developed Ekman spirals are rarely observed in narrow and sheltered valleys, in the very broad plain east of Granite Peak relatively low Rossby numbers, and hence an impact of rotational effects, are conceivable.

Part of the variability in *N* below 4000 m above m.s.l. is related to the occasional development of a deep convective boundary layer during the day. Figure 6c and e provides evidence that mixed layers ($N^2\approx 0$) grow up to an altitude of approximately 4500 m above m.s.l. (i.e., to a thickness of about 3000 m). At night, especially within 300 m of the ground, very stable layers with $N^2$ up to $8\times 10^{-4}$ s$^{-2}$ are possible.

The variability of the wind-direction profiles is less obvious. Winds from the southerly sector tend to have a consistent direction below 4000 m above m.s.l. during the day (Fig. 6d) and to be subject to stronger frictional veering at night (Fig. 6f), possibly as a consequence of the diurnal variability of stratification. The more frequent north-north-westerly winds during the day are apparent, but whether because of a playa breeze or an upvalley flow is uncertain. Rare and almost exclusively nocturnal easterlies are also apparent in Fig. 6b, d and f, probably connected to dynamically-forced flow from the western slope of Cedar Mountain, which might occasionally extend as far as the middle of the sagebrush plain, around 8–10 km from the foot of the slope.

To summarize, the 4DWX model simulations suggest that aspects of the PBL structure at Dugway Proving Ground are largely determined by predictable mesoscale circulations generated by the topography or by land-surface inhomogeneities. However, especially during the night, interaction with the dominant synoptic flow regimes (e.g., southerly or north-westerly winds) appears to be responsible for additional phenomena, such as flow diversion around topography and dynamically-accelerated downslope flow, mostly confined to the northern or north-eastern flanks of mountains.

Results presented herein provide a compact summary of the prevalent wind regimes at the Dugway Proving Ground area, but cannot elucidate the respective forcing factors thoroughly. Conclusive evidence for the driving mechanisms of the different prevailing winds can most likely be obtained only through a detailed study of their diurnal and seasonal variations.

In the following section we investigate whether the bottoms of mountain slopes around the Dugway Proving Ground are the most favourable areas, during the night, for flow separation and convergence conducive to unusually vigorous mixing.

## Nighttime Processes: Flow Separation

In Sect. 3 we formulated the hypothesis that orographically-induced modification of the ambient flow might foster low-level flow convergence and flow separation at the Dugway Proving Ground area, in particular at night when the atmosphere is stably stratified near the ground. Because the terms *convergence* and *separation* might seem unrelated to each other, or even contradictory, it is useful to clarify the definition of the latter. Boundary-layer separation occurs when a strong adverse pressure gradient force decelerates near-surface flow and eventually reverses its direction. When this occurs, mass continuity requires the flow to be lifted off the surface, hence the concept of separation (Scorer 1958; Batchelor 1967). Near-surface wind vectors on opposite sides of the separation line converge into it, hence the approximate equivalence of the terms *convergence* and *separation* in the context of the present discussion.

Boundary-layer separation is only one of many processes that might relate to flow convergence near the ground. Others include thermal updrafts developing over the crests of mountain ridges (Serafin and Zardi 2010), or even processes entirely unrelated to PBL dynamics, e.g., fronts or gravity currents. However, boundary-layer separation is the only process favouring convergence in a stable PBL that would systematically occur in the immediate vicinity of topographic features. This motivates the special emphasis given to this phenomenon in the present study.

Both (large-scale) dynamical forcing and (local-scale) thermal forcing can be responsible for flow separation at the bottom of a slope. Thermal forcing is primarily related to cold-air pooling, leading to a positive pressure anomaly in the region where cold air accumulates. The magnitude of the anomaly can be estimated by integrating the hypsometric equation. In fact, using $p_\mathrm{s}$ and $p_\mathrm{c}$ to refer respectively to the surface pressure in unperturbed conditions and to the surface pressure at the same location in presence of a cold pool, the following relationship can be derived,

1 $$\begin{aligned} p_\mathrm{c}=p_\mathrm{s}\exp \left\{ \frac{g{\Delta } z{\Delta } T}{R_\mathrm{d}T(T-{\Delta } T)}\right\} \end{aligned}$$

wherein ${\Delta } z$ is the depth of the cold pool and ${\Delta } T$ its intensity (in K), assumed to be constant with height up to ${\Delta } z$. For instance, taking $p_\mathrm{s}=865$ hPa (the Dugway Proving Ground lies at approximately 1300 m above m.s.l.), a strong nocturnal cold pool (e.g., ${\Delta } z=20$ m, ${\Delta } T=15$ K, $T=280$ K) would yield $p_\mathrm{c}=864.88$ ($p'=0.12$ hPa). Therefore, for realistic cold-pool depths and intensities, thermal forcing is not expected to generate pressure anomalies larger than 0.1 hPa at Dugway Proving Ground.

Dynamical forcing, on the other hand, can cause different separation regimes (e.g., bluff-body separation at mountain top or wave-induced separation) that correspond to distinct ranges of values of two non-dimensional parameters (Baines 1997; Ambaum and Marshall 2005). These are the mountain aspect ratio, $h_m/L$, ($h_m$ being the mountain height and *L* its half-width) and the upstream non-dimensional mountain height, $Nh_m/U$ (*U* being the ambient wind speed). Considering the aspect ratio of Granite Peak (approximately 0.2), both separation regimes might occur in its lee, with a preference for wave-induced separation down the lee slope in the stable nocturnal environment. Negative pressure perturbations generated over the slope by mountain waves can easily exceed a few tenths hPa (see below and Fig. 8, 9 for examples), suggesting that dynamical forcing induces separation more likely than thermal forcing in this area.

A plausible scenario for boundary-layer separation at Dugway Proving Ground is the following. Under certain conditions, ambient flow over or around obstacles in a stable environment generates mountain waves. Pressure perturbations embedded in waves of sufficiently large amplitude force the near-surface flow to separate. Separation occurs downstream of a localized pressure minimum, i.e., in a region of adverse pressure gradient force, typically near the foot of lee slopes. Air upstream of the separation point, displaced downwards by the wave, is related to a warm anomaly because of adiabatic compression heating. Extremely stable air at the bottom of the lee side of mountains, if present, hydrostatically intensifies the adverse pressure gradient generated by the wave, further favouring the tendency of the near-surface flow to separate. Large gradients of wind speed, pressure, and temperature occur along the separation line. Evidence in support of this scenario is provided below.

Detecting convergence lines related to flow separation from extensive output data from the 4DWX model system requires analyzing not only time series of near-surface wind fields, but also of pressure and temperature fields. An appropriate processing of pressure and temperature data proves to be necessary, in order to filter out their obvious altitudinal and seasonal variability. Our filtering method consists of two steps.

First, an areal average $\overline{\phi }^{xy}$ is removed from the original signal $\phi$ (a 2-year time series of either pressure or temperature at 1-h intervals). The detrended signal $\phi ''$ is defined as $\phi ''(x,y,t)=\phi (x,y,t)-\overline{\phi }^{xy}(t)$. This step essentially removes the fingerprint of synoptic systems, which cause time-dependent but approximately spatially homogeneous temperature and pressure perturbations in the relatively small Dugway Proving Ground area. The result is a set of detrended time series (one per each grid point in the 4DWX model domain) or, in other words, a map of time series.

Second, the detrended signal $\phi ''$ is subjected to high-pass temporal Lanczos filtering, yielding the filtered signal $\phi '$. The Lanczos method is a filtering approach in Fourier space that significantly reduces the Gibbs phenomenon, which appears as spurious oscillations near sharp discontinuities in the filtered series. Originally introduced into the field of meteorology (Duchon 1979), in more recent years Lanczos filtering has gained wide popularity in a number of disciplines including image processing. High-pass filtering removes from $\phi ''$ any oscillations related to seasonal or diurnal variability (a cut-off frequency of (1/12) hr$^{-1}$ was adopted in this study). The resulting filtered signal $\phi '$ only retains the fingerprint of high-frequency or intermittent atmospheric disturbances. Time series from all grid points are treated independently from each other during this stage.

**Fig. 7:**
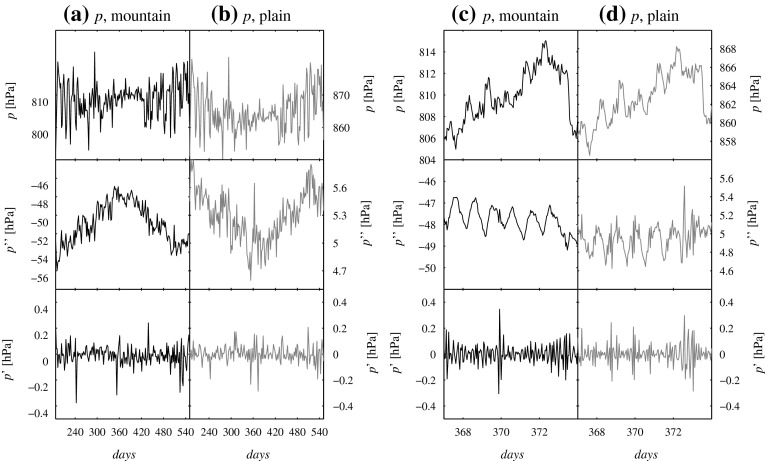
Effect of filtering on the pressure time series from 4DWX model system output. Pressure, *p*, is the original series, $p''$ the result of detrending, and $p'$ the result of Lanczos low-pass filtering. Panels **a** and **b** show the effects of filtering at annual scale. Panels **c** and **d** refer to the diurnal scale

The effects of this two-step filtering at seasonal and diurnal scales are illustrated in Fig. 7, which shows pressure time series at two grid points in the 4DWX model domain, one at the top of Granite Peak, another a few km east of it. Removal of area-wide trends from the pressure field leaves discernible altitudinal, seasonal, and diurnal signals in $p''$ (Fig. 7a–d). The pressure perturbation is negative at the mountain top and positive on the lowland. Furthermore, the pressure difference between mountain top and lowland is smaller in summer and during daytime, which can be understood from the hypsometric equation (the larger the temperature in a column, the smaller the pressure difference between two height levels). Lanczos filtering, resulting in the time series $p'$, removes all of these sources of variability. Similar reasoning is valid for the temperature series (not shown).

**Fig. 8:**
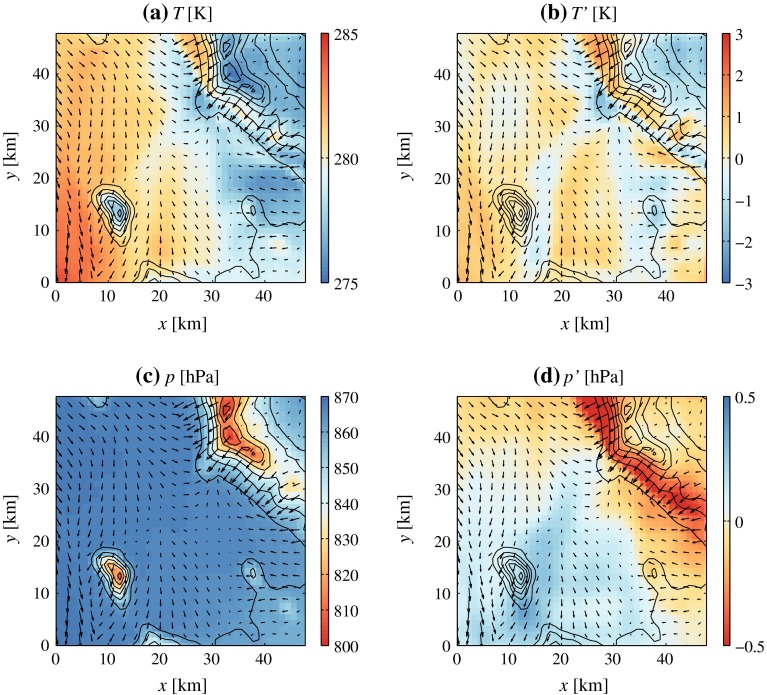
Example of (**a** and **c**) unfiltered and (**b** and **d**) filtered temperature and pressure fields in the core of 4DWX model domain 4. Vectors represent the wind field at the first model level above the ground. *Plots* refer to model output on 12 May 2014 at 0900 UTC (0200 LT)

**Fig. 9:**
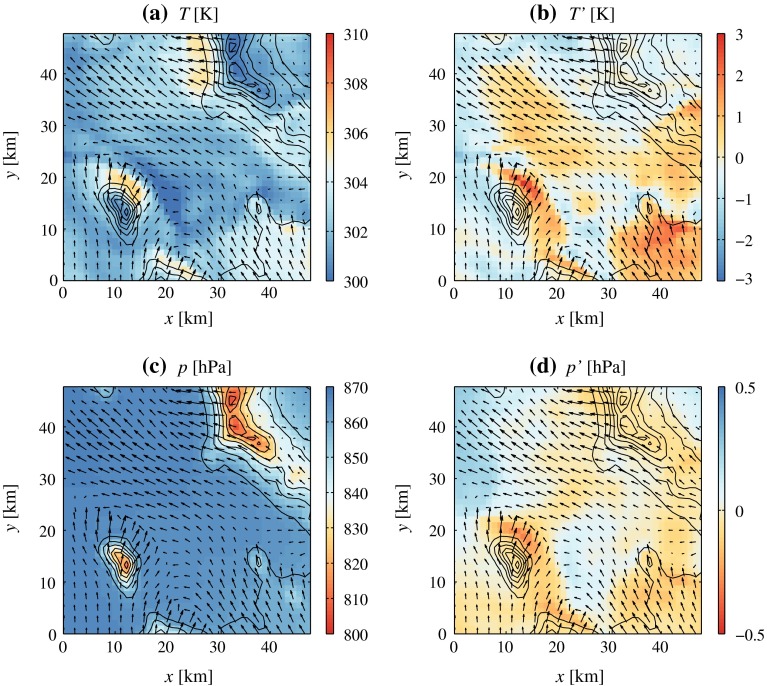
As in Fig. 8, but for 8 August 2012 at 0700 UTC (0000 LT)

Although time series from each grid point are treated independently during the Lanczos filtering step, filtered fields (i.e., maps) maintain spatial coherence, as shown in Figs. 8 and 9. The two examples show how filtering *p* and *T* data makes the distinctive features of flow separation apparent. In Fig. 8, the ambient flow is northerly, and relatively strong north-easterly downslope flow occurs on the south-west slope of the Cedar Mountains. A warm anomaly in the unfiltered *T* field is co-located with the region of high wind speeds. However, no obvious relationship is visible between the wind field and the unfiltered *p* field, which only shows a distinct altitudinal fingerprint. After detrending and filtering, the temperature contrast along the downslope flow front is intensified, while a negative pressure perturbation ($p'<-0.5$ hPa) appears on the south-west slope of the Cedar Mountains. The downslope flow reacts to the adverse pressure gradient force encountered at the foot of the slope by separating. Analogous dynamics are apparent in Fig. 9, which refers to a case with southerly ambient flow. In these conditions, dynamically-forced downslope flow occurs on the north-east slopes of Granite Peak and the Dugway Range. Even in this case, downslope flow acceleration and separation are related to localized pressure minima on the lee sides of mountains, while a strong temperature contrast is present across the separation line.

**Fig. 10:**
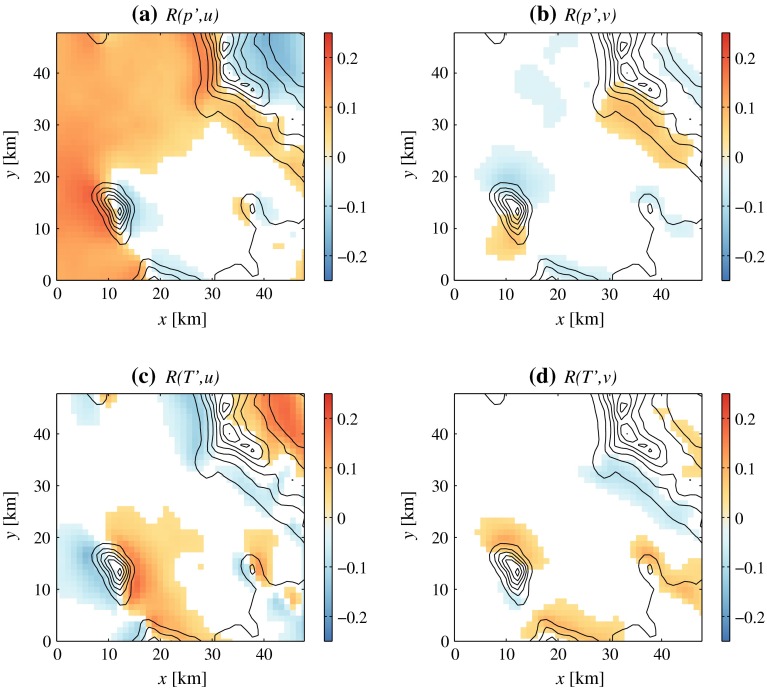
Maps of linear correlation coefficients between perturbation pressure $p'$ and *u* (panel **a**), $p'$ and *v* (**b**), perturbation temperature $T'$ and *u* (**c**), and $T'$ and *v* (**d**). *White areas* represent regions where the correlation is not statistically significant (i.e., the *p* value of the correlation is larger than 0.001)

These examples show that, when dynamical forcing is active, a certain degree of correlation between the anomalies of field variables can be expected. For instance, negative pressure perturbations coincide with positive anomalies of wind speed and temperature on the lee slopes of mountains. These correlations are apparent in long-term time series of perturbation variables ($p'$ and $T'$) and wind speed, as shown in Fig. 10, which results from the analysis of all nocturnal data in the 2-year period. Linear correlation coefficients (*R*) are computed between time series of two variables at each grid point in the 4DWX model system domain, and results are displayed as maps of *R*.

The linear correlation coefficients in the different panels of Fig. 10 never exceed 0.25. This is not surprising since the occurrence of dynamical forcing is sporadic and since the relationships between perturbation variables are not expected to be linear. Nevertheless, correlations are statistically significant and physically meaningful. Significance follows from the fact that the *p*-value (i.e., the probability of achieving an equally high *R* between two uncorrelated variables by random chance) is lower than 10$^{-3}$ in all the coloured areas in Fig. 10. Meaningfulness is determined by the fact that correlation coefficients are spatially coherent even though time series from each grid point are treated independently. Furthermore, the correlation patterns shown in Fig. 10 have a clear physical explanation.

A positive *u* (respectively *v*) wind component tends to be associated with a negative pressure anomaly (negative *R*) and with a positive temperature perturbation (positive *R*) in the lee (eastern, respectively northern) side of mountains, exactly as expected when stably stratified air flows over them. Similarly, a negative *u* (respectively *v*) wind component tends to be associated with a negative pressure anomaly (positive *R*) and with a positive temperature perturbation (negative *R*) on the western (respectively southern) sides. As a result, distinctive positive/negative patterns across mountains appear in the correlations between field variables. Interestingly, diurnal thermal forcing generates an entirely different pattern at night, at least in the absence of cold-air pooling: local pressure highs and temperature lows centred on mountains. Correlation coefficients tend to be higher around Granite Peak than near other mountains in the region. Widespread positive values of $R(p',u)$ over the playa, visible in Fig. 10a, likely reflect the approach of cold fronts from the west towards Dugway Proving Ground.

Regions of significant correlation between wind components, pressure, and temperature extend only a few km from the bottom of mountain slopes. This suggests that dynamically-forced leeside winds, causing boundary-layer separation, generate microscale fronts in those areas. Strong and approximately co-located gradients of surface wind speed, pressure, and temperature are expected to occur here. Gradient magnitude is easily computed from surface 4DWX model data at each grid point and every output time, so a climatographical evaluation of where the highest wind speed, temperature, and pressure contrasts occur at the Dugway Proving Ground area is possible. Our sample consists of $3.2\times 10^7$ elements (44 grid points along the *x* and *y* directions, and 16,588 output times). For each point in the sample, the magnitudes of the horizontal gradients of the wind speed, temperature, and pressure are computed. The 99th percentiles of the three frequency distributions are then found; $||\nabla U||_{99}$, $||\nabla T'||_{99}$, and $||\nabla p'||_{99}$ correspond respectively to 2.6 $\times$ 10$^{-3}$ s$^{-1}$, 6.3 $\times$ 10$^{-4}$ K m$^{-1}$, and 7.6 $\times$ 10$^{{-5}}$ hPa m$^{-1}$. These apparently small values are actually orders of magnitude larger than typical synoptic-scale values. Also, $||\nabla p'||_{99}$ is considerably larger than the horizontal pressure gradient that would result by integrating $||\nabla T'||_{99}$ over a reasonable depth, further supporting the idea that flow separation on lee slopes is primarily dynamically driven.

**Fig. 11:**
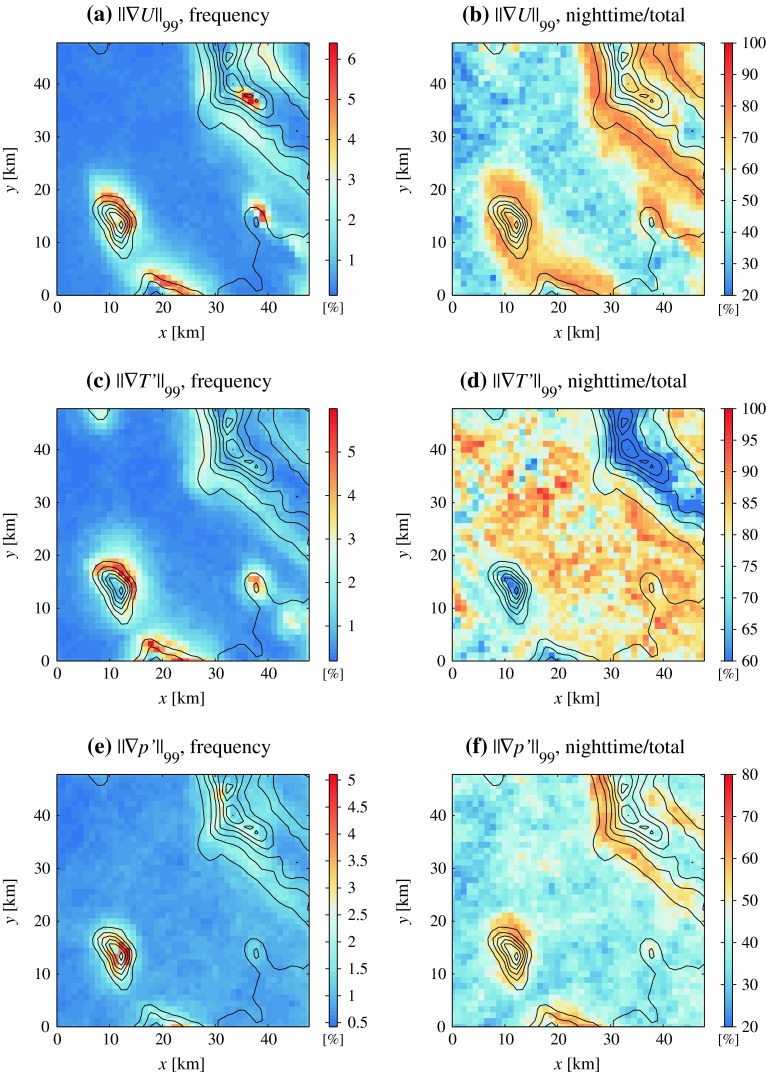
Two-dimensional histograms representing the spatial distribution of extreme values of the magnitude of the gradients of **a** wind speed, **c** filtered temperature and **e** filtered pressure. A spatially uniform distribution of extremes would correspond to a uniform value of 1. Panels **b**, **d**, and **f** represent the fraction of nighttime events with respect to the total in each bin of panels **a**, **c**, and **e**, respectively

The spatial distributions of simulated gradient magnitudes larger than $||\nabla U||_{99}$, $||\nabla T'||_{99}$, and $||\nabla p'||_{99}$ can be evaluated, and results of this elaboration are shown in Fig. 11. If extreme values were uniformly distributed over the Dugway Proving Ground domain, the frequency of exceedence of the 99th percentile would equal 1 % everywhere. Instead, there is a large degree of spatial variability.

The highest wind speed, temperature, and pressure contrasts tend to occur at the foot of mountains (*u* and *T* fields) or on the low stretches of slopes (*p* field), consistent with flow separation slightly downstream of a pressure minimum (Fig. 11a, c, e). The bulk of boundary-layer separation cases occurs during the night, as evinced by the fact that the nighttime fraction of large wind speed and pressure gradients is mostly above 50 % near mountain flanks (Fig. 11b, d, f). The north-eastern slopes of Granite Peak and the Dugway Range are preferred areas, likely because of the dominance of southerly flows in this area (see Sect. 3 above). In a nocturnal stable environment, frictional veering of southerly flows (see again Sect. 3) favours boundary-layer separation on north-east slopes; in this scenario, while low-level southerly flow is blocked, south-westerly flow at mountain-top level continues down the mountain slopes.

Separation lines along the north-east slopes of Granite Peak and the Dugway Range constitute convergence zones, where dynamically-accelerated flow on the steep mountain flanks interacts with drainage flow developing over the gently sloping plain. The resulting “collision” of air masses is expected to lead to considerable turbulence and enhanced mixing (El-Madany et al. 2014; Fernando et al. 2015).

In previous studies related to the MATERHORN field campaigns the colliding air masses were described as drainage flows, implicitly suggesting that “collisions” depend on differential cooling along slopes and adjacent valleys (e.g., Lehner et al. 2015). The results discussed above demonstrate that pressure and temperature perturbations caused by stable airflow over or around topography also have the potential to force boundary-layer separation and cause low-level convergence, preferentially at the bottom of slopes on the lee side of mountains and even in absence of diabatic effects.

## Discussion and Conclusions

Two years of simulations from a limited-area weather prediction model are analyzed in order to provide insight into yet poorly understood aspects of nocturnal PBL circulations in an area with complex topography and land cover, viz. Dugway Proving Ground in north-western Utah.

Mesoscale numerical weather prediction models are run operationally by many institutions around the world, primarily for the purpose of forecasting. In this context, long-term archives of past model simulations are normally employed for forecast verification. In contrast, examples of climatographical analysis of past operational mesoscale model data are uncommon. Some are related to the study of the spatial and interannual variability of rainfall (e.g., Hahmann et al. 2009) or to the assessment of wind-energy potential (Nawri et al. 2014; Santos-Alamillos et al. 2014).

Our study focuses instead on nocturnal PBL phenomena and aims to quantify the impact of mesoscale topography on airflow patterns. Regular thermally- or dynamically-driven circulations, which result from characteristics of the land surface (such as topography or thermal properties), have long offered the promise that mesoscale numerical weather prediction models can skillfully simulate these phenomena (Vukicevic and Errico 1990), at least in a climatological sense. We demonstrate that 4DWX model simulations realistically represent the prevalent wind directions and typical wind speeds observed at several locations across Dugway Proving Ground, as well as their diurnal variability. Based on this outcome, we rely on data from the 4DWX model system to describe the impacts of topography on the wind field in this region.

The most frequent surface wind regimes in Dugway Proving Ground correspond to southerlies and north-westerlies. In both cases, considerable veering (exceeding 45$^\circ$) is typically observed in the lowest 1 km of the atmosphere, especially at night. As expected, topography appears to modify the two basic patterns in various ways, e.g., by (a) diverting flow around obstacles, (b) promoting near-surface drainage of cold air, and (c) favouring flow acceleration on leeside slopes in certain flow regimes. In the latter case, flows often converge at the bottom of slopes, in particular north-east of mountains. These topographically-induced convergence areas display the distinctive features of flow separation (i.e., strong gradients of wind speed, temperature and pressure). Dynamically forced boundary-layer separation results in the formation of microscale convergence zones near the foot of mountains.

The plausibility of these results needs to be evaluated carefully, in view of the typical limitations of mesoscale weather models. Pointwise comparison of wind fields from 4DWX model simulations and observational data supports the conclusion that the modelled and observed wind climates are in good agreement. However, the simulations considered herein have a rather coarse resolution (1.1-km horizontal grid spacing) and rely on numerical hyperdiffusion to suppress noise in operational runs. Also, common PBL parametrization schemes have a well-known tendency to be over-diffusive in the nocturnal, very stable boundary layer (Grisogono 2010). All of these factors conspire to remove small-scale variability from solution fields and to damp spatial gradients. The absolute values of wind speed, temperature, and pressure gradients mentioned should therefore not be interpreted literally. However, since the 1.1-km grid interval is more than sufficient to characterize many of the key features of orography, there is no reason to doubt that the spatial distribution of topographically-induced phenomena in the model fields is credibly reproduced in a climatological sense.

The most likely boundary-layer separation scenario in the lee of Granite Peak at Dugway Proving Ground is far more complex than the simple conceptual model that is typically considered in laboratory, theoretical, and even numerical studies (e.g., Baines and Hoinka 1985; Vosper 2004; Ambaum and Marshall 2005; Jiang et al. 2007).

First, the leeside response to stratified flow over a mountain depends on its horizontal aspect ratio (Smith 1989). Therefore, ambient flow from different directions towards an irregularly shaped obstacle like Granite Peak may have largely different effects on the leeside boundary layer.

Also, the incoming flow may not (and in general does not) correspond to a straight hodograph, with constant wind direction at all heights. We have shown above that frictional veering in the convective boundary layer during the day, and very stable layers decoupling low-level flow from the free atmosphere during the night, are likely at Dugway Proving Ground. This implies that the diagnostic parameters normally considered in forecasting boundary-layer separation and rotors (e.g., shallow-water Froude number and non-linearity parameter; see for instance Vosper 2004, and Jiang et al. 2007) are not easily evaluated in this case, and may even be uninformative. Even without directional shear, the mere variability of wind speed and stability with height poses significant interpretation challenges (Reinecke and Durran 2008).

Finally, diabatic effects such as cold-air pooling, which are normally not taken into account when studying boundary-layer separation, might to some extent affect the phenomenon. From a dynamical perspective, this implies that the tendency of the boundary layer to separate from the ground may depend not only on the pressure gradient force, as commonly assumed, but also on buoyancy. To our knowledge, the impact of buoyancy effects on boundary-layer separation has received only marginal attention thus far.

As a concluding remark, we suggest that the methods used herein—the elaboration of climatographies of wind-direction distribution, extreme wind-speed distribution charts and vertical atmospheric profiles; the high-pass filtering of pressure and temperature fields; and the study of spatial variability by considering gradient maps—are general sufficient to be easily applied in other contexts, provided that an equally extensive archive of mesoscale simulations is available.
